# Etymologia: *Sporothrix schenckii*

**DOI:** 10.3201/eid2509.ET2509

**Published:** 2019-09

**Authors:** Fábio P. Sellera, Carlos E. Larsson

**Affiliations:** Universidade de São Paulo, São Paulo, Brazil

**Keywords:** Sporothrix schenckii, fungi, fungal infections, sporotrichosis, pathogenic fungus, saprophyte, zoonotic diseases, Benjamin Schenck

## *Sporothrix* [spor′o-thriks] *schenckii*

From the Greek *sporotrich* and later from the Latin *spor-* (spore) + *thrix* (hair), *Sporothrix schenckii* was named as a tribute to Benjamin Schenck, a medical student at the Johns Hopkins Hospital, who first isolated the fungus from a patient who had lesions on the right hand and arm in 1896 (Figure). This fungus was erroneously assigned to the genus *Sporotrichum* until 1962, when it was reclassified as *Sporothrix*.

**Figure:**
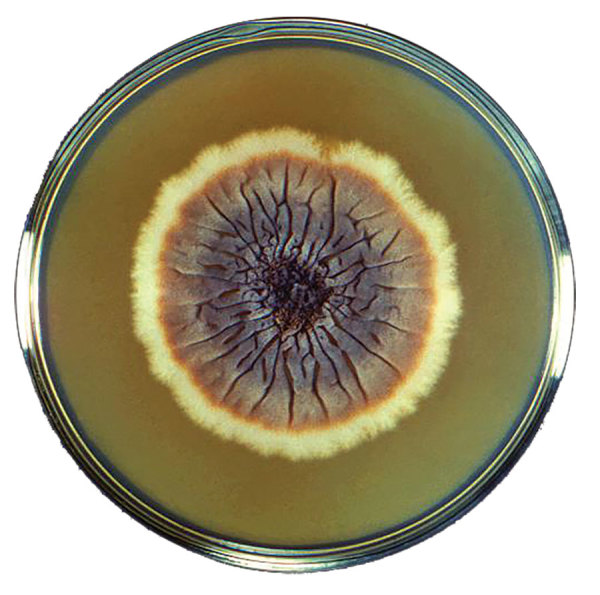
Petri dish culture of a colony of the fungus *Sporothrix schenckii* strain M-36-53. This fungus is the cause of sporotrichosis. Centers for Disease Control and Production, Dr. Lucille K. Georg, 1964.

*S. schenckii* is a saprophyte and pathogenic fungus that is responsible for sporotrichosis that is endemic mostly to tropical and subtropical regions. Sporotrichosis (also known as “rose gardener’s disease”) was related primarily to agricultural workers who had cuts or abrasions in the skin, and later to scratches and bites from companion and wild animals. Currently, it is recognized that *S. schenckii* is a species complex that includes *S.* *brasiliensis*, *S. globosa*, *S. mexicana*, *S. luriei*, and *S. schenckii sensu stricto*.
